# Insights into Fanconi Anemia Based on Molecular and Clinical Characteristics: A Multicentre Study of 13 Patients

**DOI:** 10.3390/children12080973

**Published:** 2025-07-24

**Authors:** Simoni Saranti, Nikoletta Selenti, Christalena Sofocleous, Joanne Traeger-Synodinos, Antonis Kattamis, Vassilios Papadakis, Evgenios Goussetis, Charikleia Kelaidi, Anna Paisiou, Sophia Polychronopoulou, Lydia Kossiva

**Affiliations:** 1 Second Department of Pediatrics, Medical School, National and Kapodistrian University of Athens, “P&A Kyriakou” Children’s Hospital, 11527 Athens, Greece; simsara@med.uoa.gr; 2 Laboratory of Medical Genetics, School of Medicine, National and Kapodistrian University of Athens, “Agia Sophia” Children’s Hospital, 11527 Athens, Greece; nselenti@med.uoa.gr (N.S.); csofokl@med.uoa.gr (C.S.); jtraeger@med.uoa.gr (J.T.-S.); 3 Division of Pediatric Hematology/Oncology, First Department of Pediatrics, National and Kapodistrian University of Athens, “Aghia Sophia” Children’s Hospital, 11527 Athens, Greece; ankatt@med.uoa.gr; 4 Marianna V. Vardinoyanni-ELPIDA Oncology Unit, Department of Pediatric Hematology-Oncology (TAO), “Agia Sofia” Children’s Hospital, 11527 Athens, Greece; vpapadak@otenet.gr (V.P.); ch.kelaidi@paidon-agiasofia.gr (C.K.); sophpol@otenet.gr (S.P.); 5 Stem Cell Transplant Unit, “Agia Sofia” Children’s Hospital, 11527 Athens, Greece; e.gousetis@paidon-agiasofia.gr (E.G.); salamanca.creta@yahoo.gr (A.P.)

**Keywords:** bone marrow failure, aplastic anemia

## Abstract

**Background**: Fanconi Anemia (FA) is a rare disorder, characterized by chromosomal instability, congenital abnormalities, progressive bone marrow failure, and predisposition to cancer. FA is caused by pathogenic variants in any of the 23 (*FANCA-FANCY*) linked genes. **Procedure**: Retrospective analysis of 13 FA patients with a causative variant was performed. Patients (6 boys and 7 girls) aged from 9 to 26 years old, (mean age of 7.3 years), at diagnosis. **Results:** Phenotype evaluation demonstrated in 11/13 patients’ congenital anomalies, with pigmentary changes and short stature, present in 90% of cases. Hematological abnormalities were present in 10/11 patients, with thrombocytopenia being the prominent finding. Genetic analysis for the most common complementation group FA-A revealed that 12/13 patients belonged to this group and only one patient was found to be FA-E. Exon deletions, single nucleotide variations, and duplications were identified. Familial patterns, due to consanguinity, were evident in one case. Twelve patients underwent hematopoietic stem cell transplantation (HSCT), with variable pre-HSCT supportive treatments. Post-HSCT data showed that 9 out of 10 patients for whom follow up data was available, survived for a median time of 5.4 years. Complications like acute graft-versus-host disease were noted. **Conclusions**: Our study highlights the importance of genotype towards tailored monitoring for children and families with FA.

## 1. Introduction

Fanconi Anemia (FA) is a rare chromosome instability disorder first described in 1927 by the Swiss pediatrician Guido Fanconi, in a family with congenital anomalies, anemia, recurrent infections, and spontaneous hemorrhages [1]. FA, also characterized by a predisposition to malignancies, primarily acute myeloid leukemia and head and neck squamous cell carcinomas, is the most prevalent inherited bone marrow failure syndrome (IBMF). In Europe, the prevalence ranges from 4 to 7 cases per million live births, affecting slightly more males than females [2]. Higher incidences are reported in Afrikaners, Spanish Romanis and Ashkenazi Jews, owing to the high rates of consanguinity in these populations [3,4,5].

The clinical manifestations of FA vary widely, with symptoms appearing at different ages and varying in severity. Major clinical anomalies include pigmentary changes short stature, and skeletal abnormalities such as hypoplastic or supernumerary thumbs and ocular findings like microphthalmia [6,7,8].

Hematologic abnormalities are diverse and typically manifest by the age of 7 yrs [9]. These include cytopenias, bone marrow failure (BMF) with a significantly increased risk of progression to myelodysplastic syndrome (MDS), or acute myeloid leukemia (AML). According to data from the International Fanconi Anemia Registry (IFAR), the cumulative incidence of BMF by the age of 40 is 90% with a median age of onset of 7.6 years [10]. Additionally, FA patients have a heightened risk of solid tumours, particularly head and neck squamous cell carcinoma [11]. Endocrine dysfunction, growth retardation, and structural anomalies in various organs such as the central nervous system, kidneys, ears, heart, and gastrointestinal tract are also frequently observed. Additionally, reduced fertility is a common feature, with almost all male and approximately half of female patients being infertile [12,13].

FA is caused by pathogenic variants in any of the 23 related genes (*FANCA-FANCY*) with *FANCA*, *FANCC*, and *FANCG* being responsible for 80% of cases. Dysfunctional FA/BRCA pathway results in DNA damage and genomic instability, leading to abnormal chromosomal rearrangements and disruptions of the cell cycle.

Diagnosis typically involves the cytogenetic detection of chromosome breaks and the molecular analysis of *FANC* genes currently performed via Next Generation Sequencing techniques. Understanding genotype–phenotype correlations is crucial for managing FA and developing new therapeutic strategies [14].

Allogeneic hematopoietic stem cell transplantation (HSCT) remains the sole curative therapy for bone marrow failure in FA patients. However, challenges such as donor availability and treatment toxicity exist. Supportive therapies like transfusions, androgens, cytokines, and thrombopoietin receptor agonists are also utilized [15,16,17]. Gene therapy efforts primarily focus on the FA-A subtype, while there are also emerging advancements in gene editing techniques [18,19,20].

A previous cohort of patients with Fanconi Anemia treated at our institutions was focusing on genotypic data, and clinical features, and outcomes, including genotypic data [21,22]. The current study reports on additional patients with genotypic data in this rare disease. The aim of this study was to identify possible correlations between genotype, phenotypic characteristics and disease deterioration, through the retrospective analysis of patients’ records along with the assessment of disease treatment options and outcomes.

## 2. Materials and Methods

This retrospective multicentre observational study included 13 patients followed by the Hematology Unit–Second Department of Pediatrics, National and Kapodistrian University of Athens, “P. and A. Kyriakou” Children’s Hospital, the Pediatric Hematology and Oncology Unit First Department of Pediatrics, National and Kapodistrian University of Athens, “Aghia Sophia” Children’s Hospital, the Bone Marrow Transplantation Unit, and the Department of Pediatric Hematology and Oncology of the “Aghia Sophia” Children’s Hospital, Athens, Greece. Both Hospitals are referral centres for pediatric patients living in central and southern Greece.

Four of the thirteen patients were included from a previously published clinical cohort of 20 children with Fanconi anemia and bone marrow failure, diagnosed between 1985 and 2015 at the Department of Pediatric Hematology and Oncology, Aghia Sophia Children’s Hospital (Table 1: Patients 4, 5, 6, and 12) [22]. Their genotypic characteristics were previously reported [21].

As this was a retrospective study, no additional genetic testing was performed. Data were collected from existing molecular diagnostic reports issued by the Laboratory of Medical Genetics (LMG), National & Kapodistrian University of Athens, since 2009. All patients were initially evaluated with chromosome breakage test using Diepoxybutane. According to the reports, DNA had been isolated from peripheral blood lymphocytes, and molecular analysis of exons 1–43 of the *FANCA* gene was performed. The techniques applied included PCR (Polymerase Chain Reaction) followed by direct sequencing for the detection of sequence variants, and MLPA (Multiplex Ligation-dependent Probe Amplification) for the identification of exon deletions or duplications. In one of the 13 patients, Clinical Exome Sequencing had been previously conducted, and the variants identified were subsequently confirmed using the above techniques. Anonymous data from patients’ records were collected, including demographic information (age, gender), personal and family medical history along with phenotypic characteristics, genotype, treatment, disease deterioration, and hospitalization data (month and year of admission). These data were extracted from patient records stored at the Laboratory of Medical Genetics archive and were analyzed to correlate molecular findings with clinical information [20,22]. HSCT indications included, in principle, severe bone marrow failure (BMF) defined as bone marrow aplasia or transfusion need; significant isolated cytopenia (absolute neutrophil count ≤ 0.5 G/L, hemoglobin ≤ 8 g/dL, or platelet count ≤ 30 G/L); significant dysplasia/MDS without or with excess blasts; poor risk cytogenetic abnormality; AML, according to Peffault de Latour and Soulier [23]. However, in some cases, pre-emptive HSCT was performed in patients with moderate BMF defined by the presence of mild cytopenias, no siginificant dysplasia, and +1 q, −20 q, −11 q, −5 q, or −Y as a sole chromosomal abnormality. Outcomes following HSCT with variable pre-HSCT supportive treatments were assessed for 12 eligible patients. The study received ethical approval from the Ethics and Deontology Committee of the Medical School of Athens University, the Scientific Council of “Aghia Sophia” Children’s Hospital, and the Scientific Council of “P. & A. Kyriakou” Children’s Hospital and was also in accordance with the Helsinki Declaration.

## 3. Results

### 3.1. Patient Demographics

The cohort includes 13 patients, comprising seven girls (53.8%) and six boys (46.2%) aged 9 to 26 years, with a mean age of 7.3 years (SD = 3.92) at diagnosis. Phenotypic characteristics were available for 11 out of 13 patients (84%), of whom 10 (90%) had at least one congenital abnormality. The diagnosis was made following referral from treating physicians due to phenotypic features and hematological manifestations as listed in Table 1. The genotype distribution was as follows: 12 out of 13 patients harboured variants affecting the FA-A complementation group, and 1 patient in the FA-E group. Of the 13 patients in this study, 7 were Caucasian Greek and 6 were of Roma descent. Fifteen percent (15%) of the patients were offsprings of consanguineous parents. A summary of clinical characteristics of the 13 patients is shown in Table 2.

### 3.2. Congenital Abnormalities

Among 11 patients for whom clinical/phenotypic information were available, 10 had at least one congenital anomaly. The most frequent was pigmentary changes, including café au lait macules (81%), followed by short stature (height < 3rd percentile) (63%), distinctive facial features (45%), microcephaly (36%), and upper limb abnormalities (hypoplastic thumbs, radial anomalies, phalangeal dysplasia) (27%). Esophageal atresia was reported in 2 out of 11 patients.

Two out of the eleven patients met the VACTERL-H criteria (presence of at least any three of eight features) with patient 6 presenting with oesophageal atresia, annular pancreas, radial duplication, and an hypoplastic thumb and patient 9 with duodenal atresia, upper limb anomalies, and cardiac structural anomalies, whereas none of the patients met the PHENOS (pigmentation, small head, small eyes, central nervous system, otology, short stature) criteria (presence of ≥4/6 features).

### 3.3. Hematology and Malignancies

Hematological abnormal findings were present in 10 out of 11 patients at diagnosis, with thrombocytopenia being the most common cytopenia (9/11), followed by leukopenia (7/11), neutropenia (7/11), and anemia (5/11). All patients for whom data were available, presented with bone marrow failure (impaired hematopoiesis with blood cytopenias and decreased bone marrow cellularity). Regarding the development of myelodysplastic syndrome (MDS), one patient developed MDS at the age of 6 years with a complex bone marrow karyotype and subsequently developed non-Hodgkin lymphoma. The time-period from the first hematological manifestation of the disease until the referral to a tertiary hospital ranged from several months to a few years.

### 3.4. Types of FA Gene Variants

Based on the patients’ molecular analysis reports, detection of causative variants involved the molecular analysis of *FANCA* gene via direct Sanger Sequencing and MLPA (Multiplex Ligation Dependent Probe Amplification) for all 43 exons and adjacent areas. One case underwent Clinical Exome Sequencing identifying variants subsequently confirmed with standard techniques.

In this cohort, 12 patients were classified in the FA-A complementation group, and one patient to the FA-E group. Variants comprised exon deletions (5/13), single nucleotide variations (SNVs, 8/13), and nucleotide duplications (3/13) (Table 1). A detailed summary of these detected variants is provided in Table 3.

From the family history, patient 1 had a paternal grandfather who died of lymphoma at the age of 39, a paternal grandmother with short stature, skeletal abnormalities, and renal failure, as well as a maternal grandmother with short stature. Patients 5 and 6 are dizygotic twins, both carrying deletions of exons 1–5 and 7–17 of the *FANCA* gene. Patients 8 and 9 are siblings born to consanguineous parents (second cousins). Patients 10 and 11 are also siblings, compound heterozygous for a deletion of exons 1–30 of the *FANCA* gene and an c.82G>T; p. (Gly28*) variant in exon 2, initially misevaluated as homozygous due to the trans deletion.

### 3.5. Treatment and Outcome

Twelve of the thirteen (12/13) patients underwent hematopoietic stem cell transplantation (HSCT). Nine out of twelve (9/12) HSCTs were performed at the Bone Marrow Transplant Unit of “Agia Sophia” Children’s Hospital. The mean age of patients undergoing HSCT at the Bone Marrow Transplant Unit was 9.4 years, with an average time of 2.5 years from diagnosis to HSCT. The longest interval was observed in a patient diagnosed within the first year of life due to bilateral hypoplastic thumbs, distinctive facial features, and short stature, who received HSCT at the age of 7 years and 2 months. Five out of nine who have been transplanted at the same centre had received a transplant from a non-related volunteer matched donor, while the remaining 4/9 from a related matched donor.

Details regarding conditioning, GVHD prophylaxis, and donor matching were available for nine patients (patients 1, 2, 5, 6, 7, 8, 9, 11, and 13). All patients received reduced-intensity conditioning regimens, predominantly fludarabine and cyclophosphamide (Flu + CY); specifically, patients 2 and 5 received busulfan (BU) in addition to Flu/CY. GVHD prophylaxis consisted of a combination of anti-thymocyte globulin (ATG), cyclosporine (CSA), and methotrexate (MTX), administered according to institutional protocols. Donor type (volunteer unrelated or related matched donor) and GVHD occurrence are indicated in Table 1.

Pre-HSCT treatment was available in 6 patients and included transfusions (23%), granulocyte colony-stimulating factor (G-CSF) (7.6%), and corticosteroids (15.3%). The diversity in pre-HSCT treatments represents the complexity of managing FA and the individualized approach needed for these patients.

Survival data for the patients post-HSCT were available for 10 out of the 13 patients for a median follow up time of 10.32 ± 3.07 years, among whom 9 are alive and 1 is deceased. The deceased patient (4) exhibited bone marrow dysplasia, with cytogenic abnormalities del (5q14), del (17p11.2), developed MDS, underwent HSCT, and subsequently succumbed due to non-Hodgkin lymphoma [21]. Among the nine patients who underwent HSCT, three had developed acute graft-versus-host disease (GVHD) that affected only the skin (2, 5) and in one patient (1) except from the skin the gastrointestinal tract was affected as well.

## 4. Discussion

The present study provides a comprehensive analysis of 13 Fanconi Anemia (FA) patients and highlights key aspects of FA, including its genetic diversity, phenotypic manifestations, treatment options and outcomes. The findings offer insights into the genotype–phenotype correlations, the challenges in managing FA, and the efficacy of current treatment modalities.

The majority of patients in this cohort (92%) were evaluated for the presence of FA-A complementation group and turned out to belong to this group and this is consistent with previous reports [22,23]. The range of variants included single nucleotide variants (SNVs), and exonic deletions.

Almost all patients in this cohort (90%), had at least one congenital abnormality with pigmentary changes being the most common. This is in line with the established phenotypic spectrum of FA, where such abnormalities serve as critical diagnostic indicators [29,30]. Additionally, two out of the eleven patients (18%) met the VACTERL-H criteria (**V**ertebral, **A**nal, **C**ardiac, **T**racheo-esophageal fistula, **E**sophageal atresia, **R**enal, upper **L**imb and Hydrocephalus) (OMIM 192350), a prevalence consistent with previous reports [22,31]. VACTERL-H and PHENOS (skin Pigmentation, small **H**ead, small **E**yes, **N**ervous system, **O**tology, **S**hort stature) combination may be recorded in FA. No one of the patients of the study group had such a combination or PHENOS phenotype.

As far as hematological manifestations are concerned, 11 patients (84%) developed BMF, which is in accordance with that reported in larger, multi-ethnic studies [29,30,31,32]. There is a link between the telomere length and the severity of hematological manifestations in patients with FA. In detail, the shorter the telomere length the more severe the cytopenias. The short telomeres result in accelerated apoptosis of peripheral monocytes of patients with FA. Two main mechanisms responsible for telomere shortening in FA have been reported. The first one is the high turnover of hematopoietic stem and progenitor cells, as to overcome the increased apoptosis observed in FA. Second, a direct breakage of the telomeric sequences might contribute to telomere shortening [33,34].

Allogeneic hematopoietic stem cell transplantation (HSCT) remains the cornerstone of FA treatment. Diverse pre-HSCT therapies include transfusions, G-CSF, corticosteroids, and cyclosporine. The timing of HSCT should be carefully weighed due to the fact that the procedure per se carries a significant mortality risk and especially in patients with FA it is linked with increased susceptibility to late malignancies [10,17]. In accordance with the established indications for HSCT, the patients of our study group underwent HSCT when they progress from mild to severe cytopenia, or when they were found to be positive for cytogenetic aberrations with poor prognosis or when overt MDS/AML occurred. The reduction in the doses of alkylating agents and irradiation in combination with the use of fludarabine and T-cell depletion strategies, resulted in improvement of the engraftment and decreased the GVHD rates. These findings are consistent with outcomes following HSCT in the subgroup of pediatric patients with FA and known genotypes from our previous study, further supporting the interaction between time, HCT, and genotype [22]. Indeed, improved post-HSCT outcomes in recent years have been reported in patients with comprehensive disease characterization, including systematic genotyping for FA-related genes during the same period.

In our study, 12 out of 13 patients underwent HSCT, with a median of 2.5 years from diagnosis to HSCT and a survival rate of 90% among those with available follow-up data. The occurrence of acute graft-versus-host disease (GVHD) affecting mainly the skin or the skin and the gastrointestinal tract was reported in 33% of the patients post-HSCT and this percentage is consistent with previous reports [28,35]. Our patients exhibited only acute GVHD that was treated accordingly and are still in good clinical condition.

When it comes to molecular analysis of the FA patients and the severity and course of the disease, according to the presence of *FANCA* variants, *FANCA:* c.3445_3448dup variant is a frameshift variant resulting in a premature stop codon and a truncated protein (241 aa shorter).The missing N-terminal region of the protein, is necessary for the interaction between *FANCA* and Fanconi Anemia Associated Protein 20 (FAAP20), the stability of whose is essential for the proper function of FA/BRC pathway and the accurate response to crosslink damage. Notably, c.3445_3448dup variant is common in unrelated Roma patients residing in the Balkans, possibly due to a founder effect [36]. In our study patients 8 and 9 are offsrpings of consanguineous Roma parents sharing the same aforementioned frameshift variant (*FANCA:* c.3445_3448dup) and they had a rather uncomplicated post-HSCHT course without acute GVHD.

It is also worth mentioning that siblings, such as patients 5 and 6 and patients 8 and 9, presented with notable clinical heterogeneity, despite carrying the same genetic variants [22]. Notably, patients 5 and 6 are dizygotic twins and compound heterozygotes for deletions of exons 1–5 and 7–17 of the *FANCA* gene. Patient 5 presented with a milder phenotype characterized by short stature, microcephaly, distinct facial features, and skin pigmentation abnormalities. Conversely, her twin brother, patient 6, exhibited all these features along with the VACTERL-H association (≥3/8 characteristics). From a hematological perspective, both siblings exhibited bone marrow failure. Patient 5 presented with severe aplastic anemia (Hgb = 5.6 g/dL, Plts = 24 × 10^3^/μL) and mild neutropenia, while patient 6 showed anemia (Hgb = 8.9 g/dL), moderate neutropenia (ANC = 900/μL), and mild thrombocytopenia. Both underwent HSCT from unrelated matched donors. Patient 5 underwent transplantation two years earlier than her twin brother and developed acute GvHD of the skin. Both mutations are categorized as loss-of-function (LOF) variants, as they lead to the disruption of essential coding regions required for the production of a functional *FANCA* protein. These deletions eliminate critical functional domains within the *FANCA* protein, impairing its ability to participate in the FA core complex formation, which is required for efficient DNA repair via the FA/BRC pathway. LOF mutations such as these have been strongly associated with severe phenotypic outcomes due to the inability of the truncated or absent *FANCA* protein to stabilize and interact with other core complex proteins, such as *FAAP20* and *FANCG*, both necessary for monoubiquitination of *FANCD2/FANCI* [31,36].

Several reports describe different phenotypes among siblings despite the presence of the same variants [37,38]. This suggests that a patient’s phenotype can be altered by multiple factors, beyond the underlying genetic variations, such as different genetic backgrounds, the impact of modifying genes, and environmental factors. Reversible mosaicism, where cells with the variant coexist with cells in which the variant has been spontaneously corrected, is another implicated factor that may result in a milder phenotype among relatives sharing the same molecular defect [36]. These findings underscore the genetic diversity within the FA-A complementation group among the study population. Moreover, familial segregation of clinical symptoms, particularly the early-onset lymphoma and the skeletal abnormalities as recorded in the grandparents of patient 1, suggest a possible correlation with FA, and highlight the importance of comprehensive family studies in elucidating the genetic background of FA.

The presence of LOF variants is associated with worse prognosis of the disease [31]. This highlights the importance of expanding diagnostic approaches beyond chromosome breakage tests to molecular confirmation and identification of causative variants. This is demonstrated by the more severe clinical presentation of patients 4 and 9, who carry the loss of function variants c.2T>C and c.3445_3448dup in the *FANCA* gene, respectively. More specifically, patient 4 is a compound heterozygote for the point mutations c.2T>C; P.Met1 in exon 1 and c.3788_3790delTCT; P.Phe1263del in exon 38 of the *FANCA* gene. The nonsense mutation c.2T>C; P.Met1 affects the start codon. This is a LOF mutation, and in various databases, it is classified as pathogenic based on ACMG criteria (PM3, PS1, PM2, PVS1, PS3, PP1, PP5). The c.3788_3790delTCT mutation in exon 38 is also classified as pathogenic (PM3, PM2, PM4, PM1, PS3, PP1, PP5), and it is the most common variant of *FANCA* [24,25,26,27]. It leads to the deletion of the amino acid phenylalanine at position 1263, which affects the functionality of the *FANCA* protein.

Another important finding, as far as the correlation between genotype and phenotype is concerned, was that patient 3, who was classified within the FA-E complementation group, demonstrated the earliest onset of bone marrow failure when compared to patients harbouring mutations in the *FANCA* gene.

Furthermore, molecular diagnosis plays a crucial role in the long-term management of these patients. Understanding the molecular basis of the disease allows clinicians to predict and promptly manage potential complications that may arise over time. Regular monitoring of disease progression through hematological parameters and periodic screening for solid tumours is essential for timely intervention, when necessary, especially in patients belonging in the FA-D1 and FA-N complementation groups.

Among the limitations of the study, is the small sample size that limits the interpretation of the findings and increases the risk of selection bias while, the retrospective nature of the study, whereby phenotypic characteristics are extracted from the hospital records, may result in incomplete or inconsistent data. Most patients in this study had targeted molecular analysis for *FANCA* variants (as was available in the initial molecular genetic analysis at first referral) and only one was analyzed using Clinical Exome Sequencing currently included in the molecular tests applied to all new referrals. The exclusion of patients tested elsewhere or with incomplete clinical data further increases the risk of selection bias in this cohort. These limitations highlight the need for the establishment of big national registries with the participation of all the referral centres for FA.

## 5. Conclusions

This study provides insights into the genetic and clinical landscape of Fanconi Anemia in a cohort of Greek patients. The findings underscore the importance of genotype–phenotype correlations in understanding disease progression and highlight the efficacy and challenges of current treatment strategies. The timing of HSCT is very important and should be ideally offered before the deterioration of the disease. The diversity of variants within the *FANC* related genes calls for comprehensive genetic screening to tailor individualized monitoring, as patients with loss of function mutations might need more intensive surveillance resulting in timely HSCT, due to poor prognosis. Moreover, the diversity of the clinical manifestation and the hematological deterioration among siblings with the same molecular defect highlights the need for evaluation towards a possible presence of mosaicism. The patients suffering from FA are in need of close monitoring and timely intervention. Establishing national and international registries for Fanconi Anemia patients is highly important for improving patient care and research on the field of this rare genetic disorder.

## Figures and Tables

**Table 1:** Data regarding the gender, age at diagnosis, congenital anomalies and hematological manifestations, chromosome breakage analysis result, complementation group, variants found after molecular testing, whether patients underwent HSCT, with a Related Matching Donor or a Voluntary Unrelated Donor and complications of the transplant. A: Male, F: Female, Y: Height, BMF: Bone Marrow Failure, Hgb: Hemoglobulin, Plts: platelets, ANC: Absolute Neutrophil Count, HSCT: Hematopoietic Stem Cell Transplantation, GvHD: Graft Versus Host Disease, RMD: Related Matched Donor, VUD: Volunteer Unrelated Donor, CALM: café au lait macules, FTT: Failure to thrive. Patients 4, 5, 6, and 12 were reported in Kelaidi et al. [22]. Pairs of siblings were noted as Sib1 (A and B), Sib2 (A and B) and Sib3 (A and B).

	Sex	Consanguinity	Ethnicity	Age at Diagnosis (Years)	Congenital Anomalies	Hematologic Manifestations	Chromosome Breakage Analysis	Complementation Group	Variants	Carrier Parents
1	F	NO	Greek	5.5	Short stature, failure to thrive, delayed bone age, multiple CALM	BMF from the age of 5.5 years	Positive	FA-A	c.449G>T: p.Glu150* c.2633G>C: p.E878Q	Yes
2	M	NO	Greek	4	Autistic traits, neurodevelopmental delay	Thrombocytopenia, leukopenia	Positive	FA-A	ex.9-29del IVS20-1G>A	Yes
3	F	NO	Greek	4	None	BMF from the age of 3 years	Positive	FA-E	c.1313T>C; p.Leu438Cys	Yes
4	M	NO	Greek	5	Low weight (<3rd percentile) dysmorphic facial features, microcephaly, nail dystrophy	BMF from the age of 4.5 years, MDS with del(5q14), del (17p11.2), non-Hodgkin lymphoma	Positive	FA-A	c.2T>C: p.Met1? c.3788_3790delTCT:p.Phe1263del.	Yes
5 (Sib1A)	F	NO	Gypsy/Roma	6	Short stature (<3rd percentile) dysmorphic facial features, microcephaly, skin hyperpigmentation	BMF (severe aplastic anemia) with Hgb = 5.6 g/dL, Plts = 24 (10^3^/μL) and neutropenia (ANC = 1300/μL), bone marrow hypoplasia	Positive	FA-A	ex. 1-5del ex. 7-17del	Yes
6 (Sib1B)	M	NO	Gypsy/Roma	6	Short stature (<3rd percentile) dysmorphic facial features, microcephaly, ptosis, undescended testicle, oesophageal atresia, annular pancreas, radial duplication, hypoplastic thumb, skin hyperpigmentation	BMF, Hgb = 8.9 g/dL, neutropenia (ANC = 900/μL) and mild thrombocytopenia (Plts = 111 × 10^3^/μL), bone marrow hypoplasia	Positive	FA-A	ex. 1-5del ex. 7-17del	Yes
7	F	NO	Gypsy/Roma	11	FTT, short stature (<3rd percentile), CALM	BMF from the age of 12 years Bone Marrow Karyotype: +chromosome 6	Positive	FA-A	c.3445_3448dup	Yes
8 (Sib2 A)	F	YES	Gypsy/Roma	10	Face asymmetry, ptosis (right), strabismus, CALM, 2 and 3 toe syndactyly, lumbar scoliosis, midline brain cyst	BMF from the age of 10.5 years	Positive	FA-A	c.3445_3448dup	Yes
9 (Sib2 B)	M	YES	Gypsy/Roma	5	FTT, delayed bone age, duodenal atresia, CALM, hypogonadism	BMF from the age of 5.5 years	Positive	FA-A	c.3445_3448dup	Yes
10 (Sib3A)	M	NO	Greek	16	Uknown	Uknown	Uknown	FA-A	c.82G>T: p.Gly28* ex. 1-30del	Yes
11 (Sib3B)	F	NO	Greek	12	Uknown	Uknown	Uknown	FA-A	c.82G>T: p.Gly28* ex. 1-30del	Yes
12	M	NO	Gypsy/Roma	9	Short stature (<3rd percentile) microcephaly, thumb dysplasia with epiphyseal dysplasia of the nail phalanx and hypoplasia of the phalanx	BMF from the age of 10.5 years	Positive	FA-A	in. 2 (IVS02 +1G>A c.1304G>A: p.Arg435His	Yes
13	F	NO	Greek	1	Short stature (<3rd percentile) dysmorphic facial features, CALM, thumb dysplasia	BMF from the age of 6.5 years	Positive	FA-A	c.3521G>A; p.Trp1174* c.3788_3790delTCT: p.Phe1263del	Yes

**Table 2:** Characteristics of 13 patients with Fanconi Anemia. Listed are the gender, median age at diagnosis and HSCT, congenital anoma-lies, hematologic disorders, and therapeutic interventions. HSCT: Hematopoietic Stem Cell Transplantation, GvHD: Graft Versus Host Disease, RMD: Related Matched Donor, VUD: Volunteer Unrelated Donor, G-CSF: Granulocyte Colony Stimulating Factor, ATG: Anti-Thymocyte Globulin.

Variable	N = 13 (%)
Sex	
Male	6 (46.2)
Female	7 (53.8)
Mean age at Diagnosis	7.3 years
Mean age at HSCT	9.4 years
Congenital abnormalities	
Unknown	2 (15.3)
None	1 (7.6)
Any anomaly	10 (77)
Types of congenital anomalies	
Unknown	2 (15.3)
Skin pigmentation disorders	9 (69.2)
Failure to thrive	8 (61.5)
FA facies	5 (38.4)
Skeletal abnormalities	5 (38.4)
Microcephaly	4 (30.7)
Gastrointestinal tract	2 (15.3)
Eyes	2 (15.3)
Genitourinary anomalies	2 (15.3)
CNS structure	1 (7.7)
Endocrine disorders	2 (15.3)
Hematologic Manifestations	
Bone Marrow Failure	
Unknown	2 (15.3)
Yes	11 (84.6)
No	0
Hematologic Malignancies	
Unknown	2 (15.3)
Yes	1 (7.6)
No	10 (77)
Cytopenia	
Unknown	2 (15.3)
Thrombocytopenia	9 (69.2)
Anemia	5 (38.4)
Neutropenia	7 (53.8)
Therapies before HSCT	
Unknown	7 (53.8)
None	3 (23)
Yes	3 (23)
Transfusions	3 (23)
G-CSF	1 (7.6)
Corticosteroids	2 (15.3)
Cyclosporine	1 (7.6)
ATG	1 (7.6)
HSCT	
Unknown	1 (7.6)
No	0
Yes	12 (92.3)
No information about the HSCT	3 (23)
RMD	4 (30.7)
VUD	5 (38.4)
aGVHD	3 (23)

**Table 3:** Summary of the detected variants. Class.: Classification, P: Pathogenic, V: Variant of Unknown Significance, NA: Not Available, Ref: References.

Case No.	Sex	Affected Gene	Mutation 1 cDNA/Protein	Class.	Allele Frequency in Population Databases (gnomAD)	Ref.	Mutation 2 cDNA/Protein	Class.	Allele Frequency in Population Databases (gnomAD)	Ref.
1	F	FANCA	c.449G>T; p.Glu150*	P	0.00%	NA	c.2632G>C; p.Glu878Gln	P	0.00%	[24,25]
2	M	FANCA	ex.9-29del	P	NA	NA	IVS20 -1G>A	P	NA	NA
3	F	FANCE	c.1313T>C/;p.Leu438Cys	V	0.00%	NA	c.1313T>C; p.Leu438Cys	V	NA	NA
4	M	FANCA	c.2T>C; p.Met1?	P	0.018%	[21,26]	c.3788_3790delTCT; p.Phe1263del.	P	0.018%	[27]
5 (Sib1A)	F	FANCA	ex. 1-5del	P	NA	NA	ex. 7-17del	P	NA	NA
6 (Sib1B)	M	FANCA	ex. 1-5del	P	NA	NA	ex. 7-17del	P	NA	NA
7	F	FANCA	c.3445_3448dup	P	<0.001%	[28]	c.3445_3448dup	P	<0.001%	[28]
8 (Sib2 A)	F	FANCA	c.3445_3448dup	P	<0.001%	[28]	c.3445_3448dup	P	<0.001%	[28]
9 (Sib2 B)	M	FANCA	c.3445_3448dup	P	<0.001%	[28]	c.3445_3448dup	P	<0.001%	[28]
10 (Sib3A)	M	FANCA	c.82G>T: p.Gly28*	P	0.00%	[26]	ex. 1-30del	P	NA	NA
11 (Sib3B)	F	FANCA	c.82G>T; p.Gly28*	P	0.00%	[26]	ex. 1-30del	P	NA	NA
12	M	FANCA	in. 2 (IVS02 +1G>A)	P	NA	[21]	c.1304G>A; p.Arg435His	P	<0.001%	[27]
13	F	FANCA	c.3521G>A; p.Trp1174*	p	<0.001%	[26]	c.3788_3790delTCT; p.Phe1263del	P	0.018%	[27]

## Data Availability

The original contributions presented in this study are included in the article. Further inquiries can be directed to the corresponding author.

## Abbreviations

FA	Fanconi Anemia
HSCT	Hematopoietic stem cell trasplantation
MDS	Myelodysplastic syndrome
BMF	Bone marrow failure
IFAR	International Fanconi Anemia Registry
AML	Acute myeloid leukemia
LMG	Laboratory of Medical Genetics
SNVs	Single nucleotide variants
FAAP20	Fanconi Anemia Associated Protein 20
VACTERL-H	**V**ertebral, **A**nal, **C**ardiac, **T**racheo-esophageal fistula, **E**sophageal atresia, **R**enal, upper **L**imb and **H**ydrocephalus
PHENOS	Pigmentation, small head, small eyes, central nervous system, otology, short stature
GVHD	Graft versus host disease
LOF	loss-of-function
Plts	Platelets
ANC	Absolute neutrophil count
